# Effect of Diamond-Coated Ultrasonic Tip Finishing on Resin Cement Bond Strength to Dentin

**DOI:** 10.1590/0103-644020266906

**Published:** 2026-08-07

**Authors:** Leonardo Alvares Sobral Silva, Giovanni de Souza Goulart Cardoso, Lucas Tadeu Pereira dos Santos, Clarisse Maria Luiz Silva, Matheus de Paula Costa, Milton Santamaria, João Maurício Ferraz da Silva

**Affiliations:** 1 Institute of Science and Technology, São Paulo State University (Unesp), São José dos Campos, São Paulo, Brazil; 2 Departamento de Odontologia Social e Pediátrica, Instituto de Ciência e Tecnologia, Universidade Estadual Paulista (Unesp), São José dos Campos, Brasi; 3 Department of Dental Materials and Prosthodontics, Institute of Science and Technology, São Paulo State University (Unesp), São Jose dos Campos, Brazil

**Keywords:** Dentin, Resin cement, Dental finishing, Bond strength, Shear strength, Dentina, Cimento resinoso, Acabamento dental, Resistência de união, Resistência ao cisalhamento

## Abstract

This study aimed to evaluate the effect of dentin surface finishing protocols using ultrasonic CVD diamond tips on the bond strength of resin cement to dentin. Seventy-two bovine incisor crowns were prepared and assigned to four dentin surface finishing protocols (Groups 1-4, n = 18), including a conventional control group and experimental groups using ultrasonic CVD diamond tips applied sequentially with increasing fineness. One specimen per group was analyzed by scanning electron microscopy to assess surface topography and smear layer characteristics. The samples were bonded using light-cured resin cement in combination with three adhesive strategies: total-etch, self-etch, and universal adhesives. After 24 hours of storage in distilled water, microshear bond strength testing was performed (0.5 mm/min). Data were analyzed using two-way ANOVA and Tukey's post hoc test (α = 0.05). Groups 3 and 4 showed significantly higher bond strength than the control group (p < 0.05), while no significant differences were observed among adhesive strategies (p > 0.05). Surface analysis revealed that these protocols produced a more homogeneous and regular smear layer, whereas intermediate finishing resulted in a more irregular surface. It can be concluded that the sequential use of ultrasonic CVD diamond tips with increasing fineness improves the bond strength of resin cement to dentin and promotes a more favorable substrate for adhesion.

## Resumo

O objetivo deste estudo foi avaliar o efeito de protocolos de acabamento da superfície dentinária utilizando pontas diamantadas ultrassônicas CVD sobre a resistência de união do cimento resinoso à dentina. Setenta e duas coroas de incisivos bovinos foram preparadas e distribuídas em quatro protocolos de acabamento da superfície dentinária (Grupos 1-4, n = 18), incluindo um grupo controle convencional e grupos experimentais utilizando pontas diamantadas ultrassônicas CVD aplicadas sequencialmente com aumento progressivo da granulação. Um espécime por grupo foi analisado por microscopia eletrônica de varredura para avaliação da topografia superficial e das características da smear layer. As amostras foram cimentadas com cimento resinoso fotopolimerizável em combinação com três estratégias adesivas: condicionamento total, autocondicionante e adesivo universal. Após 24 horas de armazenamento em água destilada, foi realizado o teste de resistência de união ao microcisalhamento (0,5 mm/min). Os dados foram analisados por ANOVA de dois fatores e teste post hoc de Tukey (α = 0,05). Os Grupos 3 e 4 apresentaram valores de resistência de união significativamente maiores em comparação ao grupo controle (p < 0,05), enquanto não foram observadas diferenças significativas entre as estratégias adesivas (p > 0,05). A análise de superfície revelou que esses protocolos produziram uma smear layer mais homogênea e regular, enquanto o acabamento intermediário resultou em uma superfície mais irregular. Conclui-se que o uso sequencial de pontas diamantadas ultrassônicas CVD com aumento progressivo da granulação melhora a resistência de união do cimento resinoso à dentina e promove um substrato mais favorável à adesão.

**Figure:**
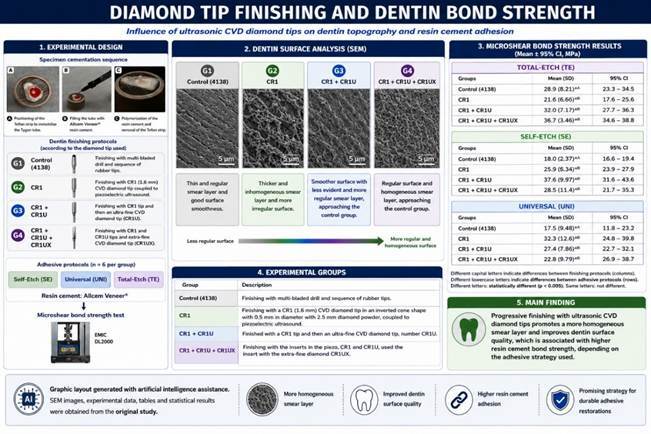

## Introduction

The development of adhesives and resin cementation, along with the introduction of ceramics in dentistry, has improved bonding to dental substrates, enabling the design of tooth structure preparations for reinforcement and rehabilitation with full crowns, partial restorations such as inlays/onlays, and ceramic laminates ^1^. A precise and successful long-term result of aesthetic restorations is related to the durability of the adhesive bond, the quality and quantity of remaining tooth structure, which contribute to improved internal adaptation, of the restoration with respect to the characteristics of the tooth preparation, as well as the mechanical characteristics of the restorative material used ^2^.

Adhesion in dentistry results from a physical and/or mechanical interaction between the adhesive and the tooth through the action of intermolecular forces. Several factors favor this attraction of molecules, such as the high surface energy of the substrate, low surface tension, and the adhesive's wetting capacity of dentin and surface preparation ^3^. Proper tooth preparation is essential for the success of adhesive restorations and requires careful technique. Among the fundamental principles for ensuring mechanical, biological, and aesthetic performance, the importance of dentin surface texture is particularly evident ^4^.

Recent studies report that the smoothness and finish of the preparation influence seating and retention of indirect restorations cemented with the resin cementation technique ^5^. Furthermore, it improves laboratory procedures, impression taking, and the quality of the working model. These characteristics are achieved through finishing and polishing the surface of the preparation, which is crucial for bonding, as it reduces contamination and the presence of a compacted layer (the smear layer), increasing the surface energy of both the tooth and the adhesive, and decreasing the contact angle, favoring diffusion and wetting ^6^.

Currently, there are different methods for performing tooth preparation: conventional (rotary instruments), laser, and ultrasonic devices ^7^. Conventional diamond burs for high- or low-speed use are the most widely used instruments in dentistry for preparing the dental substrate, as they are easily used for enamel and dentin grinding when refrigerated, are new, and have full grinding power. However, they have limitations regarding particle loss during sterilization, which reduces cutting effectiveness, and are also relatively durable. The size of the bonded diamond particles directly affects the grinding efficiency and the smoothness of the resulting surface. The larger the particle size, the more effective the grinding, and the lower the smoothness. The smaller the particles, the less they will grind, but the smoother the surface ^7^.

Alternatives to high-speed dentistry have been researched for years ^8^. Among them, ultrasonic instruments stand out as a less invasive technique, coupled with diamond tips with characteristics distinct from those of conventional diamond tips. Most ultrasonic devices currently available on the market use piezoelectric technology, which is a property of certain crystals (such as quartz or special ceramics) that generate mechanical vibrations when subjected to an electric current. In dentistry, these crystals are used as transducers to convert electrical energy into high-frequency mechanical microvibrations (typically 25-35 kHz), enabling precision, control, and safety in clinical procedures ^9^.

Combined with piezoelectric ultrasound, dental diamond tips produced by Chemical Vapor Deposition (CVD) stand out as innovative devices for dental preparations. These tips are produced by chemical vapor deposition of a single layer of diamond strongly bonded to a molybdenum rod, with the resulting diamond able to withstand the oscillatory movements generated by ultrasound ^10^. The superiority of tips using this type of deposition technology was demonstrated by Macedo et al. ^11^ when compared to conventional diamond tips, in terms of durability, finish quality, ease of cleaning, ensuring tooth contact only with the diamond, high wear resistance, reduced pressure on the tooth structure toward the active tip, and selective wear pattern ^10^.

According to Ren et al. ^5^, polishing methods applied to the previously prepared dental substrate enhance the bonding effectiveness of self-adhesive and self-etching resin cements. The use of diamond tips chemically deposited by vapor deposition, combined with piezoelectric technology, produces a thinner, less dense smear layer, optimizing the surface texture of the tooth preparation and providing a valid alternative for improving bond strength ^12^ ^,^ ^13^.

In this context, efficient alternatives using oscillating instruments can be used for finishing preparations, aiming to improve the quality and precision of dentin grinding and thereby enhance bond strength. Based on this assumption, different tooth surface finishing protocols are proposed to improve the adhesion of the bond to dentin with resin cement. The null hypothesis was that dentin-finishing protocols using ultrasonic diamond tips would not affect the bond strength of resin cement to dentin, regardless of the adhesive strategy.

## Material and methods

### Specimen preparation

To prepare the specimens, 72 bovine maxillary incisors were obtained from Frigorífico Mantiqueira, São José dos Campos, SP. The teeth were cleaned, and the periodontal ligament fibers were removed with a No. 15 scalpel blade. Subsequently, an endodontic opening was performed with a No. 3 round bur (KG Sorensen, Brazil) to remove the pulp chamber roof completely. The root portion of all teeth was removed by transverse section along the long axis of the tooth using a carborundum disk. Subsequently, the enamel surface was reduced with a plaster reamer to expose the dentin layer. Using a caliper, the remaining dentin thickness was standardized at 0.8 mm. Subsequently, all crowns were reduced with a 4138 diamond bur to a thickness of 0.5 mm. The specimens were placed in a silicone matrix for embedding in chemically activated acrylic resin (Jet-Clássico, Brazil), with the vestibular surfaces of the teeth exposed. The specimens were stored for 24 hours in distilled water at 37°C, and then the dentin surface was finished according to each group described in Table 1. After finishing, the teeth were stored in distilled water for another 24 hours. Each group initially consisted of 18 specimens. One specimen from each group was randomly selected for SEM analysis. The remaining specimens were allocated to bond-strength testing, with six assigned to each adhesive strategy.

**Table 1:** Division of groups according to the type of diamond tip used to perform tooth preparation

Group	Description
1	Control. Finishing with a multi-bladed drill and a sequence of rubber tips;
2	4138 + CR1: Finishing with a CR1 (1.6 mm) CVD diamond tip in an inverted cone shape with 0.5 mm in diameter, with 2.5 mm diamond powder (CVDentus, São José dos Campos - SP, Brazil), coupled to a piezoelectric ultrasound (Clinical model, CVDentus, São José dos Campos - SP, Brazil).
3	4138+CR1+CR1U: Finished with a CR1 tip and then an ultra-fine CVD diamond tip, number CR1U.
4	4138+CR1+CR1U+CR1UX: Finishing with the inserts in the piezo, CR1 and CR1U, used the insert with the extra-fine diamond CR1UX.

### Surface topography analysis by sem

Before the adhesive procedure, one specimen from each group was randomly selected (from the initial n=18) for SEM analysis to evaluate the smear layer characteristics using scanning electron microscope (SEM) (JEOL/JSM-5310, Tokyo, Japan). Each tooth was cross-sectioned into a 6-mm-diameter disc with a glass bur on a circular specimen cutter (MicroMill) under refrigeration. Afterward, these dentin specimens were processed for SEM, including fixation in 2.5% glutaraldehyde for 24 h and dehydration in increasing concentrations of ethanol (50%, 60%, 70%, 85%, 95%, and 100%) for 15 min, applied twice ^14^. Then, the dentin surfaces of the specimens were coated with metal and observed under SEM. The micromorphology of the dentin surface prepared by the separate finishing methods was observed ^15^.

### Adhesive procedures

The teeth of each type of dentin finishing protocol were divided into 3 subgroups, with 6 test specimens in each of them, divided according to the types of adhesive procedure:

Total-Etch Group - The bonding technique was performed using a 3-step adhesive (Opti Bond FL Plus), with 15 seconds of dentin etching, followed by 30 seconds of washing and air-drying of the substrate, and the application of primer and bond from separate bottles.

SE Group - The bonding technique was performed using a 2-step self-etch adhesive (Clearfil SE Bond, Kuraray).

Uni Group - The bonding technique was performed using a one-step self-etching Universal Adhesive (Single Bond Universal - 3M).

### Microshear test

After applying the adhesives, the samples were positioned in a metal device with a bipartite Teflon matrix, with a 2 mm diameter and a 2 mm-height hole. To prepare the samples for the microshear test, Tygon R-3603 tubes (Norton Performance Plastic Co.) with an external diameter of 2 mm, a height of 1 mm, and an internal diameter of 0.8 mm were used to delimit the adhesion area ^16^. Using the Teflon matrix, the Tygon tube was immobilized on the dentin. Inside these tubes, using an application syringe, the light-curing resin cement Alcem Veneer (FGM Produtos Odontológicos, Joinville, SC, Brazil) was applied in a single increment and photoactivated for 40 s to obtain small cylinders for the mechanical test.

**Figure 1:**
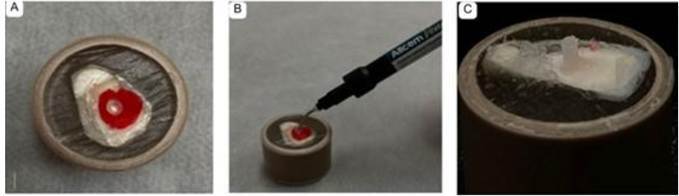
Specimen cementation sequence. (A) positioning of the Teflon strip to immobilize the Tygon tube; (B) filling the tube with Allcem Veneer® resin cement; and (C) polymerization of the resin cement and removal of the Teflon strip.

The specimen was then removed from the matrix and subjected to additional photoactivation for 40 seconds. After 1 hour at room temperature, the Tygon tube was removed using a No. 15 scalpel blade (Med Blade), taking care not to induce any stress in the composite (Figure 1). All specimens were stored in distilled water in a bacteriological oven at 37°C for 24 hours and then subjected to microshear testing. The specimens were subjected to laboratory mechanical testing to evaluate bond strength under microshear stress. This test was performed on an EMIC DL2000 universal testing machine with a 10 kg load cell. This machine has a built-in computer terminal that reads data transmitted by the mechanical test using a dedicated program. A metal base was used to allow the correct positioning of the specimens, and a 0.2 mm-diameter orthodontic archwire was attached to the upper movable end of the machine. During the test, the wire formed a loop around the composite resin cylinder and was positioned at the adhesive interface. The speed used was 0.5 mm/min. The microshear strength was determined as the ratio of the maximum force applied during the test to the area of adhesion. This value, expressed in MPa, determines the load required to break the adhesive bond at the dentin-composite resin interface ^17^.

### Statistical analysis

The data obtained were subjected to statistical analysis using Jamovi software (version 6.0, Sydney, Australia). The data were analyzed using two-way ANOVA (factors: finishing protocol and adhesive strategy), followed by Tukey’s post hoc test for pairwise comparisons when significant differences were detected (p=0.05).

## Results

### Microshear test

The mean adhesive strength values expressed in MPa ± 95% CI (confidence interval) are shown in detail in Table 2. The Shapiro-Wilk and Levene p-values ​​demonstrate that the data are normal and homogeneous (p>0.05). The columns demonstrated that the group 3 and 4 protocols required a greater load for the bond failure, with mean values of (32.0±7.17) and (36.7±3.46), respectively, with a statistical difference from the control group (p<0.05). The results of the group 2 protocol were similar to those of group 1 (p>0.05). Row comparisons showed that the bond strength values between the adhesive procedures were similar (p>0.05).

**Table 2:** Mean microshear bond strength values (±SD) of adhesive bond strength assessment.

Groups	Total-Etch (MPa)	Self-Etch (MPa)	Universal (MPa)
1	28.9 (8.21)^Aa^	18.0 (2.37)^Aa^	17.5 (9.48)^Aa^
2	21.6 (6.66)^ABa^	25.9 (3.34)^ABa^	32.3 (12.6)^ABa^
3	32.0 (7.17)^Ba^	37.6 (9.97)^Ba^	27.4 (7.86)^Ba^
4	36.7 (3.46)^Ba^	28.5 (11.4)^Ba^	32.8 (9.79)^Ba^

Means with the same letters are not significantly different by Tukey's test (p > 0.05). Different uppercase letters indicate differences in finishing protocols (columns). Different lowercase letters indicate a difference between adhesive protocols (rows).

### SEM topography analysis

**Figure 2:**
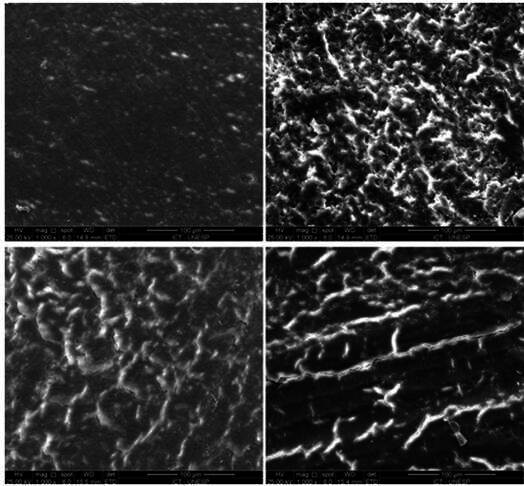
Topographic characteristics of the specimens from each experimental group, respectively. (A) Dentin surface of the control group (4138) presents good surface smoothness and an apparently thin and regular smear layer. (B) Dentin surface presenting larger and inhomogeneous irregularities. (C) The dentin surface appears smoother and with a less evident and more regular smear layer, in relation to group 2 (4138+CR1), approaching the control group. (D) Dentin surface with fewer irregularities as in figures B and C, in addition to presenting a homogeneous smear layer, approaching the control group. Source: Prepared by the author

SEM analysis of the surface morphology is shown in Figure 2, revealing differences in their topographies. The specimens prepared using the Group 1 method exhibited a smooth dentin topography, suggesting a thin smear layer (SL). Group 2 apparently demonstrated a more irregular surface, indicating a thicker SL. The other experimental groups, which used the finishing method with fine- and ultrafine-chemical-vapor-deposition technology tips (groups 3 and 4) coupled to the piezoelectric ultrasonic instrument, showed topographic features suggestive of fewer irregularities than group 2. These findings approached the gold standard finishing protocol (group 1), suggesting greater homogeneity of the smear layer on the dentin surface.

## Discussion

In the present in vitro study, dentin surface finishing protocols using ultrasonic CVD diamond tips with increasing fineness, combined with different adhesive strategies, were evaluated regarding their effect on microshear bond strength. The results demonstrated that the finishing protocol significantly influenced bond strength, with higher values observed for the sequential use of fine and ultrafine tips (Groups 3 and 4). In contrast, no significant differences were found among the adhesive strategies. Therefore, the null hypothesis was partially rejected, as the dentin finishing protocol affected bond strength, whereas the adhesive strategy did not.

The highest and most stable bond strength values were observed for Groups 3 and 4.

The adhesive systems evaluated in this study can be applied using different strategies, including total-etch, self-etch, and universal approaches ^5^. These strategies were selected to investigate their interaction with dentin surface finishing, as previous studies have suggested that surface regularization may influence bonding effectiveness. In the present study, the superior performance of the sequential finishing protocols may be associated with improved surface characteristics that favor adhesive interaction with dentin ^15^ ^,^ ^18^.

Although SEM analysis was performed to characterize dentin surface topography and smear layer features, these observations should be interpreted qualitatively and descriptively. The images suggested that using ultrasonic CVD diamond tips with increasing fineness produced more homogeneous, regular surfaces than intermediate finishing. These morphological features may help explain the improved bond strength observed. However, no direct causal relationship can be established solely based on these observations ^19^.

It is possible that the sequential finishing protocols produced a more uniform smear layer, thereby facilitating the infiltration of adhesive monomers, particularly in self-etch systems. This mechanism involves simultaneous demineralization and incorporation of the smear layer into the hybrid layer, promoting adequate substrate wetting and interaction with dentin ^20^. Such conditions are associated with improved bonding performance and may explain the higher bond strength values observed in Groups 3 and 4. Previous studies have reported that the characteristics of the smear layer are influenced by the instrumentation used during tooth preparation. Ultrasonic diamond tips manufactured using CVD technology present high wear resistance and stable cutting performance, which may contribute to more consistent surface preparation. This may reduce debris accumulation and enhance substrate permeability, favoring interaction between adhesive systems and dentin ^21^.

Conventional diamond burs may produce thicker, more irregular smear layers, which can compromise adhesive performance, particularly in self-etch and universal systems. Therefore, finishing and polishing procedures are recommended to optimize the dentin surface prior to bonding. Alternative methods, such as laser preparation, have been described. However, their bonding performance remains inconsistent. In this context, ultrasonic instruments combined with CVD diamond tips may represent a viable alternative, as their oscillatory motion can produce more regular surfaces and potentially improve bonding outcomes. The results of this study demonstrated that finishing protocols using ultrasonic CVD diamond tips yielded bond strengths comparable to or higher than those of conventional methods, regardless of the adhesive strategy used. This suggests that the effect of surface finishing may be more relevant than the choice of adhesive system under the conditions evaluated ^22^.

Universal adhesives are generally classified as mild self-etch systems, and their interaction with the smear layer is a critical factor for bonding effectiveness. In total-etch systems, the smear layer is removed during acid etching, whereas in self-etch systems, it is modified and incorporated into the hybrid layer. Although long-term degradation was not evaluated in this study, previous evidence suggests that self-etch approaches may provide more stable bonding over time than total-etch systems ^15^.

The findings of this study also reinforce that the finishing protocol influences the thickness and uniformity of the smear layer. Qualitative SEM observations suggested that finer diamond tips produced more regular dentin surfaces, which may facilitate adhesive interaction. However, further studies are necessary to quantitatively assess these morphological characteristics and their direct relationship with bond strength ^23^ ^,^ ^24^.

Surface finishing and polishing procedures play a critical role in optimizing the dentin substrate for adhesion ^24^. In resin cements, particularly those with self-etching or self-adhesive properties, a thinner and more uniform smear layer may facilitate the penetration of acidic monomers and improve bonding effectiveness. However, these materials may also be susceptible to hydrolytic degradation over time due to their hydrophilic components, which were not evaluated in the present study ^25^.

Despite the limitations inherent to an in vitro study, including the use of bovine teeth, the results indicate that ultrasonic CVD diamond tips may represent a promising approach for dentin surface finishing. Their use may improve bond strength and more consistently achieve surface characteristics. Further clinical studies are recommended to evaluate the long-term performance of these protocols under clinical conditions ^25^.

## Conclusion

Within the limitations of this in vitro study, it can be concluded that dentin surface finishing with CVD diamond-coated ultrasonic tips, used with piezoelectric devices, significantly improves the bond strength of light-cured resin cement to dentin. In addition, these finishing protocols promote a more homogeneous and uniform smear layer, which appears to favor adhesive performance. Therefore, the sequential use of fine and ultrafine ultrasonic diamond tips represents a promising approach to optimize dentin surface preparation and enhance adhesion in indirect restorative procedures.

## Data Availability

The research data are available upon request.
